# Revealing extracellular electron transfer mediated parasitism: energetic considerations

**DOI:** 10.1038/s41598-017-07593-y

**Published:** 2017-08-10

**Authors:** Roman Moscoviz, Clément Flayac, Elie Desmond-Le Quéméner, Eric Trably, Nicolas Bernet

**Affiliations:** 0000 0001 0360 9610 grid.419083.7 LBE, INRA, Univ Montpellier, 102 Avenue des étangs, 11100 Narbonne, France

## Abstract

Extracellular electron transfer (EET) is a mechanism that allows energetic coupling between two microorganisms or between a microorganism and an electrode surface. EET is either supported by direct physical contacts or mediated by electron shuttles. So far, studies dealing with interspecies EET (so-called IET) have mainly focused on possible syntrophic interactions between microorganisms favoured by this mechanism. In this article, the case of fermentative bacteria receiving extracellular electrons while fermenting a substrate is considered. A thermodynamical analysis based on metabolic energy balances was applied to re-investigate experimental data from the literature. Results suggest that the observations of a decrease of cell biomass yields of fermentative electron-accepting species, as mostly reported, can be unravelled by EET energetics and correspond to parasitism in case of IET. As an illustration, the growth yield decrease of *Propionibacterium freudenreichii* (−14%) observed in electro-fermentation experiments was fully explained by EET energetics when electrons were used by this species at a potential of −0.12 ± 0.01 V vs SHE. Analysis of other cases showed that, in addition to EET energetics in *Clostridium pasteurianum*, biological regulations can also be involved in such biomass yield decrease (−33% to −38%). Interestingly, the diminution of bacterial biomass production is always concomitant with an increased production of reduced compounds making IET-mediated parasitism and electro-fermentation attractive ways to optimize carbon fluxes in fermentation processes.

## Introduction

Electromicrobiology is an emerging field that investigates the mechanisms of electron exchange between microorganisms and electrode materials^1^. So far, up to ninety-four bacterial species called electroactive bacteria (EABs) have been identified as able to perform such extracellular electron transfer (EET)^2^. These EABs can exchange electrons with other microbes or electrodes, either indirectly via redox mediators or directly via redox membrane proteins and nanowires^3^. A well-documented example involving mediated interspecies electron transfer (IET) is the syntrophic association of acid degrading bacteria and methanogenic archaea supported by H_2_/formate transfer during anaerobic digestion^4^. In the case of the studies dealing with direct IET, a typical procedure consists in growing *Geobacter metallireducens* in presence of ethanol as electron donor with no soluble electron acceptor. A second microorganism serves as electron acceptor while reducing compounds such as fumarate (*Geobacter sulfurreducens*) or CO_2_ (*Methanosaeta sp*.) without any electron donor apart from *G. metallireducens* ^5–7^. Although these experiments have provided a great insight into the mechanisms of IET, this kind of experimental design can only highlight syntrophic interactions because the two partners are dependent on each other.

Ecological interactions other than mutualism could also be promoted by IET in the case, for instance, where the electron-donating bacterium is only an additional electron source for the electron-accepting microorganism. Especially, this could be the case for fermentative species able to uptake extracellular electron during fermentation. Up to now, it has been shown that fermentative species such as *Propionibacterium freudenreichii* ^8^, *Propionibacterium acidi-propionici* ^9^, *Clostridium autoethanogenum* ^10^ *, Clostridium tyrobutyricum* ^11^ *, Clostridium acetobutylicum* ^12^ and *Clostridium pasteurianum* ^13, 14^ were able to uptake electrons either directly or indirectly (*i.e*. with artificial electron mediators) from a cathode in electro-fermentation experiments (*i.e*. fermentations in presence of a cathode that acts as extracellular electron donor^15, 16^). IET between fermentative species and EABs was previously reported between *Clostridium beijerinckii* and *G. metallireducens* ^17^ and *C. pasteurianum* and *G. sulfurreducens* ^18^. In all these experiments, extracellular electron uptake always resulted in a redistribution of the fermentation patterns towards the production of more reduced metabolites to ensure the intracellular redox balance. As an illustration, Emde and Schink^8^ observed that *P. freudenreichii* was able to produce nearly pure propionate from glucose when extracellular electrons were provided from a cathode. This result is remarkable as propionate production from glucose is a NADH-consuming pathway that has to be balanced with an NADH-producing pathway, *e.g*. the acetate pathway in *P. freudenreichii*. In this experiment, *P. freudenreichii* was able to regenerate NADH using cathodic electrons and optimize the propionate carbon recovery by avoiding acetate production.

In the same experiment, *P. freudenreichii* growth yield was decreased by 14% during electro-fermentation when compared to classic fermentation (0.299 and 0.348 g.g_glucose_^−1^, respectively). This result is surprising as the theoretical ATP generation for electro-fermentation was slightly higher than in fermentation (2.49 and 2.33 mol_ATP_.mol_glucose_^−1^, respectively). Interestingly, a similar decrease of the biomass yield for fermentative species uptaking extracellular electrons has been reported in several other studies dealing with *C. tyrobutyricum* (from 6.4 to 5.3 OD_600_)^11^, *C. autoethanogenum* (from 1.51 to 1.11 OD_600_)^10^ and *C. pasteurianum* (from 0.126 to 0.084 g.g_glucose_^−1^)^13^ electro-fermentations, or *C. pasteurianum* in co-culture with *G. sulfurreducens* (from 0.132 to 0.081 g.g_glycerol_^−1^)^18^. Unresolved, such diminution of biomass yield could be due to a combination of several mechanisms, such as (i) a redistribution of the metabolic products resulting in an altered production of ATP, (ii) energy losses related to the EET, (iii) biological regulations linked to extracellular redox potential that further affect anabolic reactions and, when present, (iv) artificial electron mediator toxicity (*e.g*. methyl viologen)^11^. The present article aims to explore the possibilities of non-mutualistic IET-mediated interactions that could explain subsequent reduction of the growth yield through a thermodynamical approach. By revisiting the data from the literature with a special focus on EET energetics, the case where a fermentative microorganism receives extracellular electrons was specifically considered.

## Results

### Thermodynamical approach and experimental dataset

**Figure 1:**
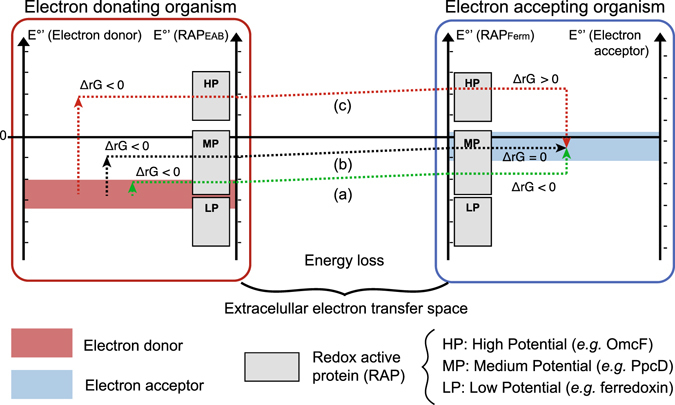
Energetic partitioning during IET: (**a**) Energetic mutualism; (**b**) Energetic commensalism; (**c**) Energetic parasitism. The ranges for redox active proteins were represented according to Santos *et al*.^22^.

In the present study, calculations were focused on the electron-accepting fermentative species, by considering the following scenario: first, electrons are supplied to such species through EET mechanisms^19, 20^ and reduce membrane-bound or intracellular redox active proteins (RAP_ferm_, *e.g*. cytochromes, ferredoxin) in the fermentative bacteria. For calculation purposes, the apparent potential at which electrons can be used by the electron-accepting bacteria will be referred to as E_RAP_, whatever the considered EET mechanism. In a second step, oxidation of these reduced RAP_ferm_ is coupled to an electron dissipation pathway where part of the fermentation substrate (*e.g*. butanol production from glucose) is used to maintain the intracellular redox balance. Depending on the E_RAP_, three possibilities concerning the reaction for electron dissipation in the electron-accepting microorganisms were considered (see Fig. 1): (a) the reaction is exergonic and can provide energy for bacterial growth (energetic mutualism); (b) the reaction does not release any free energy (energetic commensalism); (c) the reaction is endergonic and must be coupled with fermentation reactions so that the global metabolism reaction is thermodynamically favourable (energetic parasitism). In addition to this electron dissipation pathway, the fermentative species will still gain energy from fermentation reactions. Even in the case of energetic mutualism, if the electron dissipation reaction is less exergonic than fermentative catabolism, the global amount of energy available is lower than in fermentation alone, with subsequent reduction of the growth yields.

**Figure 2:**
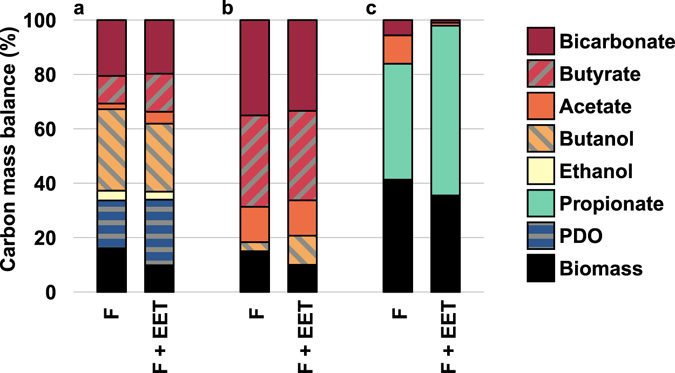
Carbon mass balances for: (**a**) Glycerol fermentation by *C. pasteurianum* in pure culture (F) and in co-culture with *G. sulfurreducens* (F + EET)^18^; (**b**) Glucose fermentation (F) and electro-fermentation (F + EET) by *C. pasteurianum* ^13^; (**c**) Glucose fermentation (F) and electro-fermentation (F + EET) by *P. freudenreichii* ^8^. Values are normalized on initial glycerol or glucose carbon content, and bicarbonate is used as adjustment variable to close the balance. The abbreviation "PDO" stands for 1,3-propanediol. The particularly high biomass yield displayed in (**c**) is likely related to the presence of yeast extract in the fermentation medium.

To assess the dependency of the growth yield on the RAP_ferm_ redox potential, three experimental datasets showing extracellular electron uptake by a fermentative species together with a decreased of the growth yields were revisited. These three cases were selected as they are the only studies dealing with electro-fermentation where complete characterization of the metabolic products as well as bacterial biomass quantification were provided. The first one concerns glycerol fermentation by *C. pasteurianum* in co-culture with *G. sulfurreducens* as electron-donating species^18^. The two other studies deal with glucose cathodic electro-fermentation by *C. pasteurianum* ^13^ and *Propionibacterium freudenreichii* ^8^. Carbon mass balances retrieved from these studies are provided in Fig. 2. In these three experiments, it could be observed that EET enhanced the production of 1,3-propanediol, butanol and propionate for the three studies, respectively. The corresponding electron dissipation pathways or net electron equivalent-consuming pathways considered for calculations were as follows:

1 $${\rm{g}}{\rm{l}}{\rm{y}}{\rm{c}}{\rm{e}}{\rm{r}}{\rm{o}}{\rm{l}}+2\cdot {{\rm{R}}{\rm{A}}{\rm{P}}}_{{\rm{f}}{\rm{e}}{\rm{r}}{\rm{m}}}^{-}+2\cdot {{\rm{H}}}^{+}\to 1,3{\mbox{-}}{\rm{p}}{\rm{r}}{\rm{o}}{\rm{p}}{\rm{a}}{\rm{n}}{\rm{e}}{\rm{d}}{\rm{i}}{\rm{o}}{\rm{l}}+{{\rm{H}}}_{2}{\rm{O}}+2\cdot {{\rm{R}}{\rm{A}}{\rm{P}}}_{{\rm{f}}{\rm{e}}{\rm{r}}{\rm{m}}}$$

2 $${\rm{g}}{\rm{l}}{\rm{u}}{\rm{c}}{\rm{o}}{\rm{s}}{\rm{e}}+4\cdot {{\rm{R}}{\rm{A}}{\rm{P}}}_{{\rm{f}}{\rm{e}}{\rm{r}}{\rm{m}}}^{-}+2\cdot {{\rm{H}}}^{+}+{{\rm{H}}}_{2}{\rm{O}}\to {\rm{b}}{\rm{u}}{\rm{t}}{\rm{a}}{\rm{n}}{\rm{o}}{\rm{l}}+2\cdot {{\rm{H}}}_{2}+2\cdot {{\rm{H}}{\rm{C}}{\rm{O}}}_{3}^{-}+4\cdot {{\rm{R}}{\rm{A}}{\rm{P}}}_{{\rm{f}}{\rm{e}}{\rm{r}}{\rm{m}}}$$

3 $${\rm{g}}{\rm{l}}{\rm{u}}{\rm{c}}{\rm{o}}{\rm{s}}{\rm{e}}+4\cdot {{\rm{R}}{\rm{A}}{\rm{P}}}_{{\rm{f}}{\rm{e}}{\rm{r}}{\rm{m}}}^{-}+2\cdot {{\rm{H}}}^{+}\to 2\cdot {{\rm{p}}{\rm{r}}{\rm{o}}{\rm{p}}{\rm{i}}{\rm{o}}{\rm{n}}{\rm{a}}{\rm{t}}{\rm{e}}}^{-}+2\cdot {{\rm{H}}}_{2}{\rm{O}}+4\cdot {{\rm{R}}{\rm{A}}{\rm{P}}}_{{\rm{f}}{\rm{e}}{\rm{r}}{\rm{m}}}$$

### Theoretical assessment for a simplified metabolism

**Figure 3:**
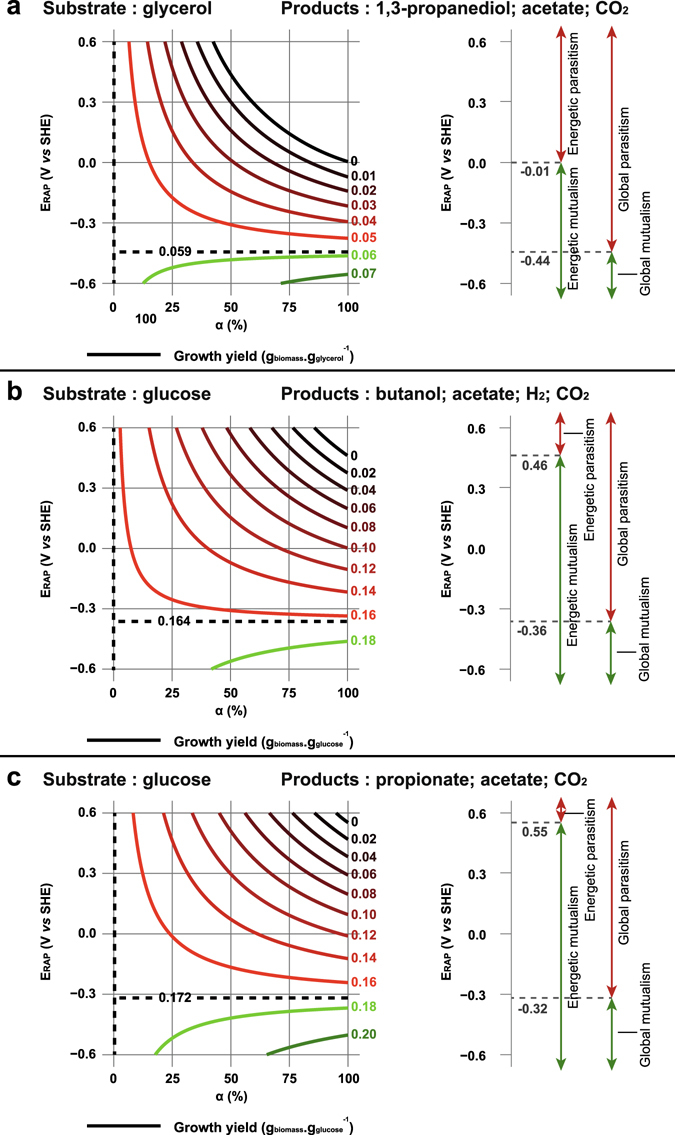
Growth yield map for a fermentative species uptaking extracellular electrons during a fermentation. (**a**) Glycerol fermentation by *C. pasteurianum*. (**b**) Glucose fermentation by *C. pasteurianum*. (**c**) Glucose fermentation by *P. freudenreichii*. Dashed lines represent contour line for the specific value obtained when α = 0 (fermentative growth yield). Solid lines represent contour lines of growth yields. These lines are green or red when higher or lower than the fermentative growth yield, respectively. Interactions are described as “Energetic” regarding energy partitioning during IET and as “Global” when biomass production of each IET partner is considered. α: fraction of substrate used for dissipating electrons from IET (normalized on substrate consumption for catabolism); E_RAP_: Potential of the redox active protein involved in the electron dissipation reaction; SHE: Standard Hydrogen Electrode.

In order to explore the impacts of EET energetics on microbial growth yields, a simplified fermentation metabolism was first considered. It comprised three metabolic reactions for each experiment: one electron dissipation pathway (equations 1–3), one reaction for minimal catabolism composed of one oxidative and one reductive pathway (*e.g*. production of acetate and 1,3-propanediol from glycerol, equations 4–6) and one biomass production reaction (anabolism, equations 7–8). The sum of the dissipation pathway and the pure fermentative catabolism was then referred to a “global catabolism” reaction in contrast with the biomass formation pathway. Using this simplified metabolism, it was possible to perform a thermodynamic state analysis as previously proposed by Kleerebezem and Van Loosdrecht^21^. This thermodynamical approach was used to determine a theoretical biomass yield based on catabolism stoichiometry and using a simple hypothesis of energy dissipation through metabolism. Indeed, the energy dissipated per gram of biomass synthetized (ΔG_dis_) was assumed to be only dependent on the elemental composition of the substrate (equation 11)^21^. Thus, growth yields were estimated as a function of E_RAP_ and the α index, a parameter indicating the fraction of fermentation substrate used for electron dissipation and normalized on substrate consumption for global catabolism. Results of this analysis are represented in Fig. 3 as a diagram showing the biomass yield contour lines as a function of E_RAP_ and α for the three selected studies.

When α is equal to 0 (*i.e*. no EET), the fermentative species biomass yields of the three cases, as calculated with the thermodynamical approach, are 0.059 ± 0.013, 0.165 ± 0.018 and 0.172 ± 0.013 g_biomass_.g_substrate_^−1^, respectively. These values are in good agreement with the growth yields calculated from theoretical ATP production for the same fermentation with 0.050, 0.145 and 0.182 g_biomass_.g_substrate_^−1^, respectively. It is theoretically possible that these biomass yields keep constant whatever the value of α in the three cases, when E_RAP_ is equal to −0.44 ± 0.01, −0.36 ± 0.01 and −0.32 ± 0.01 V *vs* SHE, respectively (see dashed lines in Fig. 3). For these very particular values of E_RAP_, the electron dissipation pathway and the minimal catabolism generate indeed exactly the same amount of energy. For lower values of E_RAP_, the electron dissipation pathway is more exergonic than the minimal catabolism and, therefore, an increased growth yield can be achieved when α > 0 (see green lines in Fig. 3). Thus, in case of IET, such situation would correspond to a mutualistic interaction between electron-donating and accepting microorganisms. For instance, H_2_/formate-mediated EET is likely to be favourable for the growth of fermentative bacteria, as the standard potential for these compounds is −0.42 V *vs* SHE (25 °C, pH 7)^20^. In contrast, higher values of E_RAP_ would be detrimental for the growth yield of the electron-accepting species at all values of α > 0 (see red lines in Fig. 3). This result clearly indicates that EET would disadvantage in most cases the growth of the fermentative partner, suggesting a kind of parasitic interaction in case of IET. An endergonic dissipation of the electrons in the three case studies (*i.e*. energetic parasitism, see Fig. 1) is also possible at E_RAP_ values higher than −0.01 ± 0.09, + 0.46 ± 0.10 and + 0.55 ± 0.06 V *vs* SHE, respectively (see Fig. 3). In this particular case, the growth can be completely stopped at high α value, as the minimal catabolism is not able to compensate the energy losses related to EET. Glucose-fermenting bacteria would unlikely be able to uptake electrons at such high potentials (> + 0.46 V *vs* SHE) under anaerobic conditions. In contrast, in glycerol fermentation, all electrons uptaken by high potential RAPs (*e.g*. OmcF, ubiquinone)^20, 22^ would make the electron dissipation pathway endergonic with subsequent drastic decrease of growth yields.

### Model parameter fitting to experimental data

As full characterization of substrate, metabolites and biomass production are provided in the three selected experimental studies, it was possible to carry out a thermodynamical state analysis using these actual experimental data. Based on these datasets and assuming that ΔG_dis_ during fermentation and fermentation + EET were equal, unique values of α and E_RAP_ were fitted for each study (see Method section and Supplementary material). The coupling parameter α calculated from experimental mass balances for the three cases is 1.34, 5.26 and 89.63% for the *C. pasteurianum* electro-fermentation, *C. pasteurianum* co-culture and *P. freudenreichii* experiment, respectively. For *P. freudenreichii*, E_RAP_ was estimated to a realistic value of −0.12 ± 0.01 V *vs* SHE, which corresponds to an exergonic electron dissipating reaction (see Fig. 1) while being a global parasitism (*i.e*. reduced growth yield). Such a potential is consistent with the use of cobalt sepulchrate as electron shuttle in this experiment (E°′ = −0.35 V *vs* SHE) and could correspond to the RAPs that have been found in *P. freudenreichii* such as menaquinone (E°′ = −0.07 V *vs* SHE) or cytochrome b (E°′ =+ 0.03 V *vs* SHE)^23^. The reduced growth yield observed in this experiment could be entirely explained by thermodynamic considerations including the metabolic shift and energy losses linked to EET. In contrast, in both *C. pasteurianum* co-culture and electro-fermentation experiments, unrealistic E_RAP_ value (*i.e*. >+ 0.5 V *vs* SHE^20, 22^) of +5.2 ± 1.0 and +22.5 ± 2.2 V *vs* SHE were required to conserve ΔG_dis_. That indicates that electron dissipation, even at low level, modified the global energy balance of *C. pasteurianum* metabolism resulting in higher ΔG_dis_ (*e.g*. increased cellular maintenance), thus reducing the growth yield. In that case, the reduction of the biomass yield could be mainly related to biological and metabolic regulations, and energetics of the electron dissipation pathway likely played a minor role.

## Discussion

Thermodynamic considerations allowed here to infer the various possible interactions (mutualist, commensal or parasitic) existing between two microbial species exchanging electrons. Depending on the electric potential of the redox active proteins (RAP), the electrons received by a fermentative microorganism can theoretically be either an additional source of energy, neutral with respect to energy or an energetic burden. By using a simple hypothesis of conservation of metabolic dissipated energy, the influence on growth yields of the RAP potential (E_RAP_) and of the fraction α of substrate used for electron dissipation was evaluated. Interestingly, these calculations indicate that energetic mutualism (*i.e*. exergonic dissipation reaction) can lead to global parasitism (*i.e*. diminution of the growth yield) when the energy gained through electron dissipation is lower than the energy generated by pure fermentative catabolism. These results are consistent with predictions of the metabolic model associated with electrosynthesis as developed by Kracke and Krömer^24^. Indeed, these authors investigated two different mechanisms of cathodic electron transport either coupled with ATP generation (Cat1 mechanism, corresponding to energetic mutualism described above) or uncoupled (Cat2 mechanism, corresponding to energetic commensalism described above). They showed that both mechanisms can result in a global mutualism through an increase of the biomass yield in the case of electrosynthesis with glucose + Cat1 mechanism, or a global parasitism for electrosynthesis with glycerol. Moreover, they analysed the example documented by Emde and Schink^8^ and concluded that Cat1 mechanism was involved. Their conclusion is consistent with a E_RAP_ value of −0.12 ± 0.01 V *vs* SHE as calculated for *P. freudenreichii* in the present study, indicating an energetic mutualism (E_RAP_ < 0.55 V *vs* SHE, cf. Figure 3C).

However, both models consider electron fluxes through only the reducing equivalents mass balance and energy balance. Therefore, experiments where only small amounts of extracellular electron uptake can cause substantial effects on the metabolic patterns could only be partly explained. As an illustration, extremely high E_RAP_ potentials would be required to explain results obtained by Moscoviz *et al*.^18^ and Choi *et al*.^13^ by energetic considerations only, with values of +5.2 ± 1.0 and +22.5 ± 2.2 V *vs* SHE, respectively. Therefore, biological regulations likely play a crucial role in such electro-fermentation phenomenon. Regulations could be related to the redox sensing and signalling mechanisms that are essential for bacteria to maintain the redox homeostasis in cells as well as adapt to different environmental redox conditions, *e.g*. in presence of oxygen^25, 26^. Harrington *et al*. (2015) showed that *Escherichia coli* could uptake electrons from a cathode using neutral red as electron shuttle^27^. They suggested that electrons were transferred to *E. coli* through the reduction of a menaquinone pool, which is known to trigger the arcB/arcA redox-sensing cascade and subsequently altered the fermentation patterns. In the case of *C. pasteurianum*, no molecular mechanism that could support similar EET has been proposed yet. Characterization of such mechanism would be necessary to better understand the effects of EET towards global metabolism.

To conclude, energetics of IET is not always beneficial for both partners and could promote interactions other than syntrophism. When a fermentative microbe is the electron-accepting partner, what could be considered as mutualism when regarding energy partitioning can still be regarded as parasitism when looking at bacterial growth. This detrimental effect can be due either (i) to a reduced energy production when fermentation metabolism is coupled to EET and/or (ii) to a metabolism modification due to biological regulations. In both cases, this reduced bacterial biomass production is concomitant with a redistribution of metabolic patterns towards a better production of highly reduced compounds (*e.g*. 1,3-propanediol from glycerol, butanol from glucose), making IET-mediated parasitism a promising way to optimize carbon recovery during fermentations. The present study also lays the groundwork for testing new hypotheses about the way for EABs to survive in a wide range of ecosystems: using fermentative species as electron sink could be an effective strategy for EABs to support their growth in the case inorganic electron acceptors are no longer available in their environment.

## Methods

### Thermodynamical state analysis for the theoretical case studies

Standard Gibbs energies (Δ_r_G°) for all chemical equations were calculated using data from Kleerebezem and Van Loosdrecht^21^. More realistic Gibbs energies values (Δ_r_G) were then estimated taking into account proton activity of 10^−7^ M (pH = 7), substrate concentrations of 50 mM for glucose and 100 mM for glycerol, and product concentrations of 10 mM except for hydrogen (3.9 10^−4^ M, corresponding to H_2_ saturation at p_H2_ = 0.5 bar).

Three different catabolic reactions were considered: fermentation of glycerol to 1,3-propanediol and acetate (equation 4), fermentation of glucose to acetate, butanol and hydrogen (equation 5) and fermentation of glucose to acetate and propionate (equation 6).

4 $${\rm{g}}{\rm{l}}{\rm{y}}{\rm{c}}{\rm{e}}{\rm{r}}{\rm{o}}{\rm{l}}\to 3/4\cdot 1,3{\mbox{-}}{\rm{p}}{\rm{r}}{\rm{o}}{\rm{p}}{\rm{a}}{\rm{n}}{\rm{e}}{\rm{d}}{\rm{i}}{\rm{o}}{\rm{l}}+1/4\cdot {{\rm{a}}{\rm{c}}{\rm{e}}{\rm{t}}{\rm{a}}{\rm{t}}{\rm{e}}}^{-}+1/4\cdot {{\rm{H}}{\rm{C}}{\rm{O}}}_{3}^{-}+1/4\cdot {{\rm{H}}}_{2}{\rm{O}}+1/2\cdot {{\rm{H}}}^{+}$$

5 $${\rm{g}}{\rm{l}}{\rm{u}}{\rm{c}}{\rm{o}}{\rm{s}}{\rm{e}}+5/2\cdot {{\rm{H}}}_{2}{\rm{O}}\to {{\rm{a}}{\rm{c}}{\rm{e}}{\rm{t}}{\rm{a}}{\rm{t}}{\rm{e}}}^{-}+1/2\cdot {\rm{b}}{\rm{u}}{\rm{t}}{\rm{a}}{\rm{n}}{\rm{o}}{\rm{l}}+2\cdot {{\rm{H}}}_{2}+2\cdot {{\rm{H}}{\rm{C}}{\rm{O}}}_{3}^{-}+3\cdot {{\rm{H}}}^{+}$$

6 $${\rm{g}}{\rm{l}}{\rm{u}}{\rm{c}}{\rm{o}}{\rm{s}}{\rm{e}}\to 2/3\cdot {{\rm{a}}{\rm{c}}{\rm{e}}{\rm{t}}{\rm{a}}{\rm{t}}{\rm{e}}}^{-}+4/3\cdot {{\rm{p}}{\rm{r}}{\rm{o}}{\rm{p}}{\rm{i}}{\rm{o}}{\rm{n}}{\rm{a}}{\rm{t}}{\rm{e}}}^{-}+2/3\cdot {{\rm{H}}{\rm{C}}{\rm{O}}}_{3}^{-}+8/3\cdot {{\rm{H}}}^{+}$$

Δ_r_G_ferm_ values for each of these reactions are −84.6 kJ/mol_glycerol_, −316.6 kJ/mol_glucose_ and −335.6 kJ/mol_glucose_, respectively.

Considering an elemental composition of the biomass as CH_1.75_O_0.5_N_0.25_^28^, the anabolic reactions for the synthesis of one C-molecule of biomass are equation 7 using glycerol and equation 8 using glucose:

7 $$2/3\cdot {\rm{g}}{\rm{l}}{\rm{y}}{\rm{c}}{\rm{e}}{\rm{r}}{\rm{o}}{\rm{l}}+1/4\cdot {{\rm{N}}{\rm{H}}}_{4}^{+}\to {\rm{b}}{\rm{i}}{\rm{o}}{\rm{m}}{\rm{a}}{\rm{s}}{\rm{s}}+1/3\cdot 1,3{\mbox{-}}{\rm{p}}{\rm{r}}{\rm{o}}{\rm{p}}{\rm{a}}{\rm{n}}{\rm{e}}{\rm{d}}{\rm{i}}{\rm{o}}{\rm{l}}+5/6\cdot {{\rm{H}}}_{2}{\rm{O}}+1/4\cdot {{\rm{H}}}^{+}$$

8 $$1/6\cdot {\rm{g}}{\rm{l}}{\rm{u}}{\rm{c}}{\rm{o}}{\rm{s}}{\rm{e}}+1/4\cdot {{\rm{N}}{\rm{H}}}_{4}^{+}\to {\rm{b}}{\rm{i}}{\rm{o}}{\rm{m}}{\rm{a}}{\rm{s}}{\rm{s}}+1/2\cdot {{\rm{H}}}_{2}{\rm{O}}+1/4\cdot {{\rm{H}}}^{+}$$

Δ_r_G_an_ values for each of these reactions are −35.5 kJ/C-mol_biomass_ and −19.0 kJ/C-mol_biomass_, respectively.

In addition, with an external input of electrons, some bacterial redox active proteins (RAP_ferm_) are reduced to RAP_ferm_^−^ and need to be oxidized back to RAP_ferm_ (cf. equations 1–3). If the potential of the redox couple RAP_ferm_/RAP_ferm_^−^ is E_RAP_ then Δ_r_G_RAP_ of these reactions are 1.0 kJ/mol_gycerol_ + 2∙F∙E_RAP_, −176.6 kJ/mol_glucose_ + 4∙F∙E_RAP_ and −212.7 kJ/mol_glucose_ + 4∙F∙E_RAP_ for equation 1, 2 and 3, respectively, with F the Faraday constant (F = 96485 C/mol).

For each case, the global catabolic reaction of the fermentative bacteria is a combination of fermentation and RAP_ferm_^−^ oxidation. For example for glycerol fermentation the global catabolic reaction is obtained by summing equations 1 and 4 multiplied by α and (1−α), respectively:

9 $$\begin{array}{c}{\rm{g}}{\rm{l}}{\rm{y}}{\rm{c}}{\rm{e}}{\rm{r}}{\rm{o}}{\rm{l}}+2\cdot \alpha \cdot ({{\rm{R}}{\rm{A}}{\rm{P}}}_{{\rm{f}}{\rm{e}}{\rm{r}}{\rm{m}}}^{-}+{{\rm{H}}}^{+})\to \\ (1-\alpha )\cdot (3/4\cdot 1,3{\mbox{-}}{\rm{p}}{\rm{r}}{\rm{o}}{\rm{p}}{\rm{a}}{\rm{n}}{\rm{e}}{\rm{d}}{\rm{i}}{\rm{o}}{\rm{l}}+1/4\cdot {{\rm{a}}{\rm{c}}{\rm{e}}{\rm{t}}{\rm{a}}{\rm{t}}{\rm{e}}}^{-}+1/4\cdot {{\rm{H}}{\rm{C}}{\rm{O}}}_{3}^{-}+1/4\cdot {{\rm{H}}}_{2}{\rm{O}}+1/2\cdot {{\rm{H}}}^{+})\\ +\,\alpha \cdot (1,3{\mbox{-}}{\rm{p}}{\rm{r}}{\rm{o}}{\rm{p}}{\rm{a}}{\rm{n}}{\rm{e}}{\rm{d}}{\rm{i}}{\rm{o}}{\rm{l}}+{{\rm{H}}}_{2}{\rm{O}}+2\cdot {{\rm{R}}{\rm{A}}{\rm{P}}}_{{\rm{f}}{\rm{e}}{\rm{r}}{\rm{m}}})\end{array}$$

with α the fraction of the global catabolism due to the oxidation of RAP_ferm_^−^ to RAP_ferm_. Thus Δ_r_G_cat_ = (−84.6 + 85.6∙α) kJ/mol_glycerol_ + 2∙α∙F∙E_RAP_.

The global metabolic reaction is a combination of this catabolism with anabolism and can be obtained by summing equations 7 with equation 9 multiplied by λ:

10 $$\begin{array}{c}(2/3+\lambda )\cdot {\rm{g}}{\rm{l}}{\rm{y}}{\rm{c}}{\rm{e}}{\rm{r}}{\rm{o}}{\rm{l}}+1/4\cdot {{\rm{N}}{\rm{H}}}_{4}^{+}+2\cdot \alpha \cdot \lambda \cdot ({{\rm{R}}{\rm{A}}{\rm{P}}}_{{\rm{f}}{\rm{e}}{\rm{r}}{\rm{m}}}^{-}+{{\rm{H}}}^{+})\to \\ 1/3\cdot 1,3{\mbox{-}}{\rm{p}}{\rm{r}}{\rm{o}}{\rm{p}}{\rm{a}}{\rm{n}}{\rm{e}}{\rm{d}}{\rm{i}}{\rm{o}}{\rm{l}}+5/6\cdot {{\rm{H}}}_{2}{\rm{O}}+1/4\cdot {{\rm{H}}}^{+}\\ +\lambda \cdot [(1-\alpha )\cdot (3/4\cdot 1,3{\mbox{-}}{\rm{p}}{\rm{r}}{\rm{o}}{\rm{p}}{\rm{a}}{\rm{n}}{\rm{e}}{\rm{d}}{\rm{i}}{\rm{o}}{\rm{l}}+1/4\cdot {{\rm{a}}{\rm{c}}{\rm{e}}{\rm{t}}{\rm{a}}{\rm{t}}{\rm{e}}}^{-}+1/4\cdot {{\rm{H}}{\rm{C}}{\rm{O}}}_{3}^{-}+1/4\cdot {{\rm{H}}}_{2}{\rm{O}}+1/2\cdot {{\rm{H}}}^{+})\\ +\,\alpha \cdot (1,3{\mbox{-}}{\rm{p}}{\rm{r}}{\rm{o}}{\rm{p}}{\rm{a}}{\rm{n}}{\rm{e}}{\rm{d}}{\rm{i}}{\rm{o}}{\rm{l}}+{{\rm{H}}}_{2}{\rm{O}}+{{\rm{R}}{\rm{A}}{\rm{P}}}_{{\rm{f}}{\rm{e}}{\rm{r}}{\rm{m}}})]\end{array}$$

Factor λ can be evaluated using a “dissipation method” as proposed by Kleerebezem and Van Loosdrecht^21^ hypothesizing that the dissipated energy associated with metabolism could be calculated as follows:

11 $$-{{\rm{\Delta }}}_{r}{G}_{met}={\rm{\Delta }}{G}_{Dis}={\rm{200}}+18\,{({\rm{6}}-NoC)}^{1.8}+\exp [{\{{(-{\rm{0.2}}-\gamma )}^{{\rm{2}}}\}}^{{\rm{0.16}}}({\rm{3.6}}+{\rm{0.4}}\,NoC)]$$

where γ and NoC stand for the oxidation state and the carbon chain length of the carbon source. For glycerol: γ = −2/3 and NoC = 3, then the expected dissipated energy is ΔG_Dis_ = 373.0 kJ/C-mol_biomass_, thus:

12 $$\lambda =\frac{-{\rm{\Delta }}{G}_{Dis}-{{\rm{\Delta }}}_{r}{G}_{an}}{{{\rm{\Delta }}}_{r}{G}_{cat}}=\frac{-337.6kJ/C-mo{l}_{biomass}}{(-84.6+85.6\,\alpha )kJ/mo{l}_{glycerol}+2\,\alpha \,F\,{E}_{RAP}}$$

And we can finally compute the yield Y_1_ (C-mol_biomass_/mol_glycerol_):

13 $${Y}_{1}=\frac{1}{\lambda \,{Y}_{S}^{cat}+{Y}_{S}^{an}}=\frac{1}{\lambda +\frac{2}{3}}=\frac{1}{\frac{-337.6kJ/C-mo{l}_{biomass}}{(-84.6+85.6\,\alpha )kJ/mo{l}_{glycerol}+2\,\alpha \,F\,{E}_{RAP}}+\frac{2}{3}}$$

Similarly yields Y_2_ (C-mol_biomass_/mol_glucose_) associated with fermentation of glucose to acetate, butanol and hydrogen (equation (2), equation (5) and equation (8)) and Y_3_ (C-mol_biomass_/mol_glucose_) associated with fermentation of glucose to acetate and propionate (equation (3), equation (6) and equation (8)) can also be computed as functions of α and E_RAP_:

14 $${Y}_{2}=\frac{1}{\frac{-217.1kJ/C-mo{l}_{biomass}}{(-316.6+140.0\,\alpha )kJ/mo{l}_{glucose}+4\,\alpha \,F\,{E}_{RAP}}+\frac{1}{6}}$$

15 $${Y}_{3}=\frac{1}{\frac{-217.1kJ/C-mo{l}_{biomass}}{(-335.6+122.9\,\alpha )kJ/mo{l}_{{glucose}}+4\,\alpha \,F\,{E}_{RAP}}+\frac{1}{6}}$$

Variations of Y_1_, Y_2_ and Y_3_ as function of α and E_RAP_ are illustrated in Fig. 3.

Values calculated above rely on the concentrations assumed for solute chemical species (pH = 7, substrate concentrations of 50 mM for glucose and 100 mM for glycerol, and product concentrations of 10 mM except for hydrogen (3.9 10^−4^ M, corresponding to H_2_ saturation at p_H2_ = 0.5 bar). To evaluate uncertainties arising from the assumptions made on these concentrations, calculations were also carried out for extreme concentration profiles that either maximize or minimize the Δ_r_G values. The first concentration profile corresponds to a “starting fermentation” with high substrates concentrations (100 mM for glucose, glycerol and ammonium) and low products concentrations (1 mM for other solute species except for hydrogen (10^−5^ M) and protons (pH = 7)). This first profile thus maximizes Δ_r_G values. The second profile corresponds to the “end of a fermentation” with low substrates concentrations (1 mM for glucose, glycerol and ammonium) and high products concentrations (100 mM for other solute species except for hydrogen (10^−3^ M) and protons (pH = 7)). This second profile, on the contrary, minimizes Δ_r_G values. Resulting maximal uncertainties are shown for yields and E_RAP_ values expressed in the main text of the article.

### Biomass yield calculation from theoretical ATP production

As metabolic pathways of glucose^24^ and glycerol^28^ have already been well characterized, it was possible to calculate theoretical ATP production for fermentations without EET. During both glucose and glycerol fermentation, ATP yields related to metabolite production could be described as follow: Y_ATP/Acetate_ = 2; Y_ATP/Propionate_ = 2; Y_ATP/1,3-propanediol_ = 0; Y_ATP/Butanol_ = 2. Considering a biomass yield of 10.5 g_biomass_.mol_ATP_^−1^ and a biomass elementary composition corresponding to CH_1.75_O_0.5_N_0.25_^28^, catabolic and anabolic reactions could be coupled. In the case of the three theoretical case studies, the ATP-balanced metabolic reactions were as follows:

16 $$\begin{array}{c}{\rm{g}}{\rm{l}}{\rm{y}}{\rm{c}}{\rm{e}}{\rm{r}}{\rm{o}}{\rm{l}}+0.045\cdot {{\rm{N}}{\rm{H}}}_{4}^{+}\to \\ 0.183\cdot {\rm{b}}{\rm{i}}{\rm{o}}{\rm{m}}{\rm{a}}{\rm{s}}{\rm{s}}+0.220\cdot {{\rm{A}}{\rm{c}}{\rm{e}}{\rm{t}}{\rm{a}}{\rm{t}}{\rm{e}}}^{-}+0.220\cdot {{\rm{H}}{\rm{C}}{\rm{O}}}_{3}^{-}+0.720\cdot 1,3{\mbox{-}}{\rm{p}}{\rm{r}}{\rm{o}}{\rm{p}}{\rm{a}}{\rm{n}}{\rm{e}}{\rm{d}}{\rm{i}}{\rm{o}}{\rm{l}}+0.372\cdot {{\rm{H}}}_{2}{\rm{O}}+0.485\cdot {{\rm{H}}}^{+}\end{array}$$

17 $$\begin{array}{c}{\rm{g}}{\rm{l}}{\rm{u}}{\rm{c}}{\rm{o}}{\rm{s}}{\rm{e}}+0.258\cdot {{\rm{N}}{\rm{H}}}_{4}^{+}+1.553\cdot {{\rm{H}}}_{2}{\rm{O}}\to \\ 1.033\cdot {\rm{b}}{\rm{i}}{\rm{o}}{\rm{m}}{\rm{a}}{\rm{s}}{\rm{s}}+0.828\cdot {{\rm{a}}{\rm{c}}{\rm{e}}{\rm{t}}{\rm{a}}{\rm{t}}{\rm{e}}}^{-}+0.414\cdot {\rm{b}}{\rm{u}}{\rm{t}}{\rm{a}}{\rm{n}}{\rm{o}}{\rm{l}}+1.656\cdot {{\rm{H}}}_{2}+1.656\cdot {{\rm{H}}{\rm{C}}{\rm{O}}}_{3}^{-}+2.742\cdot {{\rm{H}}}^{+}\end{array}$$

18 $$\begin{array}{c}{\rm{g}}{\rm{l}}{\rm{u}}{\rm{c}}{\rm{o}}{\rm{s}}{\rm{e}}+0.326\cdot {{\rm{N}}{\rm{H}}}_{4}^{+}\to \\ 1.302\cdot {\rm{b}}{\rm{i}}{\rm{o}}{\rm{m}}{\rm{a}}{\rm{s}}{\rm{s}}+0.522\cdot {{\rm{a}}{\rm{c}}{\rm{e}}{\rm{t}}{\rm{a}}{\rm{t}}{\rm{e}}}^{-}+1.044\cdot {{\rm{p}}{\rm{r}}{\rm{o}}{\rm{p}}{\rm{i}}{\rm{o}}{\rm{n}}{\rm{a}}{\rm{t}}{\rm{e}}}^{-}+0.522\cdot {{\rm{H}}{\rm{C}}{\rm{O}}}_{3}^{-}+0.651\cdot {{\rm{H}}}_{2}{\rm{O}}+2.413\cdot {{\rm{H}}}^{+}\end{array}$$

Biomass yields can directly be retrieved from these equations and are equal to 0.050, 0.145 and 0.182 g_biomass_.g_substrate_^−1^, respectively.

### Data retrieval from the literature and thermodynamical model fitting

The three case studies considered in the present article corresponded to two electro-fermentation experiments^8, 13^ and one co-culture of a fermentative microorganism with an EAB^18^. In these studies, complete characterization of metabolic products and bacterial biomass production were provided. For Emde and Schink^8^, the raw data corresponded to the results from their Table 2 for the conditions “None” (*i.e*. fermentation) and “CoS (40)” (*i.e*. fermentation + EET). In the case of Choi *et al*.^13^, data were retrieved from their Supplementary Table 2 providing electron and carbon mass balances for fermentation and fermentation + EET (referred as “control” and “BES”, respectively). Finally, data from Moscoviz *et al*.^18^ were extracted from Fig. 3 showing electron and carbon mass balances for pure culture of *C. pasteurianum* (fermentation) and co-culture of *C. pasteurianum* with *G. sulfurreducens* (fermentation + EET). Although mass balances from these datasets closed at nearly 100%, the raw stoichiometric balances were corrected using HCO_3_^−^ and H_2_ as adjustment variables to obtain a carbon and electron recovery of 100%. The stoichiometric calculations are provided as Supplementary materials and the resulting corrected carbon mass balances are displayed in Fig. 2.

To assess the model parameters α and E_RAP_ from these datasets, the amounts of substrate consumed for global catabolism and for anabolism were first estimated based on bacterial biomass quantification. In the case of Choi *et al*.^13^, bacterial biomass production during fermentation + EET corresponded to 10.4% of the initial carbon input. According to equation 8 (glucose anabolism), the molar fraction of substrate dedicated to biomass synthesis was also 10.4% in this condition, meaning that 89.6% of the initial carbon was used for global catabolism. Therefore, it was possible to calculate the α index (*i.e*. the fraction of fermentation substrate used for the electron dissipation reaction, normalized on substrate consumption for global catabolism) using quantification of the extracellular electron input. In the study of Choi *et al*.^13^, 0.2 and 99.8% of the electrons consumed by the fermentative species were provided by the cathode and glucose, respectively. According to equation 2, one mole of glucose (electron equivalent of 24)^13^ is required to dissipate 4 moles of extracellular electrons. Thus, (0.2*24)/(4*0.998) = 1.20% of initial glucose was required to dissipate these extracellular electrons. After normalizing on the substrate used for the global catabolism (*i.e*. divided by 0.896 in this case), it was found that an α value of 1.34% for this study. Similarly, α values of 5.26 and 89.63% could be retrieved from the studies of Moscoviz *et al*.^18^ and Emde and Schink^8^, respectively. In a final step, E_RAP_ could be calculated by assuming that ΔG_dis_ was equal in fermentation and fermentation + EET (see Supplementary material). For this calculation, Gibbs energies values were estimated taking into account proton activity of 10^−7^ M (pH = 7), substrate concentrations of 50 mM for glucose and 100 mM for glycerol, and product concentrations of 10 mM except for hydrogen (3.9 10^−4^ M, corresponding to H_2_ saturation at p_H2_ = 0.5 bar). Uncertainties are calculated using concentration profiles that either maximize or minimize Δ_r_G values as explained above.

## Electronic supplementary material

Supplementary Information
